# Determinants of physical activity maintenance and the acceptability of a remote coaching intervention following supervised exercise oncology rehabilitation: a qualitative study

**DOI:** 10.1007/s11764-023-01455-5

**Published:** 2023-09-21

**Authors:** Anouk T. R. Weemaes, Judith M. Sieben, Milou Beelen, Louisa T. M. A. Mulder, Antoine F. Lenssen

**Affiliations:** 1 Department of Physical Therapy, Maastricht University Medical Center+, P.O. Box 5800, 6202 AZ Maastricht, The Netherlands; 2 Department of Epidemiology, Care and Public Health Research Institute (CAPHRI), Faculty of Health Medicine and Life Sciences, Maastricht University, Maastricht, The Netherlands; 3 Department of Anatomy and Embryology, Maastricht University, Maastricht, The Netherlands; 4 Department of Human Biology, School of Nutrition and Translational Research in Metabolism (NUTRIM), Faculty of Health Medicine and Life Sciences, Maastricht University, Maastricht, The Netherlands

**Keywords:** Qualitative research, Behaviour change, Telerehabilitation, Acceptability

## Abstract

**Purpose:**

The purpose of the study was to investigate perceived determinants of physical activity (PA) maintenance following supervised exercise oncology rehabilitation and the acceptability of a remote coaching intervention during this period.

**Methods:**

A phenomenological qualitative study with semi-structured interviews was conducted. Nineteen participants (16 women, 3 men) were recruited from the intervention (*n* = 12) and control group (*n* = 7) of a randomized controlled trial on the effectiveness of remote coaching following hospital-based, supervised exercise oncology rehabilitation. Participants in the intervention group received a 6-month remote coaching intervention after completing the exercise program, aimed at stimulating PA maintenance. The interviews were based on the Capability, Opportunity, and Motivation model of Behaviour (COM-B model) and the framework of acceptability (TFA) and were coded using template analysis.

**Results:**

Key themes regarding determinants of PA maintenance were self-efficacy, PA habits, accountability, physical complaints, and facilities. Remote coaching was perceived acceptable because it stimulated PA maintenance by offering a source of structure and social support and thereby increased accountability. Moreover, it improved confidence to perform PA, leading to increased levels of self-efficacy. The remote nature of the intervention was perceived as convenient by some of the participants, while others would have preferred additional physical appointments.

**Conclusions:**

Cancer survivors considered remote coaching acceptable to stimulate PA maintenance following supervised rehabilitation. Interventions should focus on increasing accountability, self-efficacy, forming habits, and helping cancer survivors to overcome barriers.

**Implications for Cancer Survivors:**

The ability to maintain PA beyond supervised exercise oncology programs depends on many determinants. Remote coaching interventions have potential to target individually relevant determinants following exercise programs in cancer survivors.

**Supplementary Information:**

The online version contains supplementary material available at 10.1007/s11764-023-01455-5.

## Introduction

Cancer survivors can experience longstanding side effects like fatigue, declined aerobic capacity and muscle strength, psychological distress, and a diminished health-related quality of life (HRQoL) [1–5]. It has been well-established that regular physical activity (PA) improves aerobic capacity and muscle strength, reduces cancer-related fatigue and psychological distress, and consequently improves HRQoL [6, 7]. A dose–response relationship exists between post-diagnosis PA and all-cause and cancer-related mortality, with risk reductions of up to 35% [8]. Therefore, it is worrying that cancer survivors in the Netherlands spend only 34% of their waking time in physical activity and are sedentary for the remaining time [9].

Participation in a supervised exercise-based oncology rehabilitation program is a structured way to sustain or increase PA levels. However, existing literature suggests that cancer survivors experience difficulties with maintaining PA beyond the completion of a supervised exercise program [10, 11]. To sustain or increase the health benefits achieved during an exercise program, patients have to stay physically active. In a review about PA maintenance following exercise interventions, successful PA maintenance at 3 to 12 months was achieved in less than half of the included trials [12]. Schmidt et al. described in their qualitative study that cancer survivors experience the transition from a supervised hospital-based exercise program to independent community-based exercise as “a confrontation with the real world” [13].

A potential way to improve the transition phase following supervised exercise programs is by supporting it with a remote coaching intervention. Remote interventions have gained popularity and are promising in the delivery of lifestyle interventions in cancer survivors[14]. Two recent studies showed that remote interventions, like text messages and health coaching, delivered during and after a structured exercise program, are feasible and lead to increased PA levels in cancer survivors [15, 16]. Contrarily, Groen et al. reported in their meta-analysis that the effects of distance-based PA interventions in cancer survivors are small. However, no firm conclusions could be drawn from these findings, as the included trials had major limitations [17].

In order to improve PA maintenance following supervised exercise programs in cancer survivors, it is necessary to get insight into factors that influence PA behaviour during this transition period. Ferri et al. performed a qualitative study on PA maintenance 3 months after supervised rehabilitation in a tertiary hospital in Australia and reported that perceived exercise benefits motivate cancer survivors to stay active after a supervised exercise program. At the same time, the transition from a supervised environment to everyday life was a significant barrier to keep exercising [11]. When developing or refining PA maintenance interventions, it is essential to understand PA behaviour following supervised exercise programs and the context in which this behaviour occurs. Theories of behaviour change can be used to understand and unravel the underlying mechanisms [18]. The Capability, Opportunity, and Motivation model of Behaviour (COM-B model) conceptualises behaviour as part of a system of interacting factors [19, 20]. In the current study, perceived determinants of PA maintenance will be explored from the perspectives of the COM-B model.

Even when effective, implementation of interventions that support PA maintenance is only likely to succeed when these are acceptable for the target population. The Theoretical Framework of Acceptability (TFA) defines acceptability as a multi-faceted construct that reflects the extent to which people delivering or receiving a healthcare intervention consider it to be appropriate. The TFA comprises seven domains (i.e. affective attitude, self-efficacy, perceived effectiveness, ethicality, intervention coherence, burden, and opportunity costs) [21]. The TFA is considered to be helpful in assessing the acceptability of complex healthcare interventions within the development, piloting and feasibility, outcome and process evaluation, and implementation phases, as described by the Medical Research Council (MRC) guidelines [21, 22].

Dennett et al. reported that an 8-week telerehabilitation program was perceived acceptable in cancer survivors [23]. Results from Gell et al. indicate that a remote coaching intervention is acceptable to improve PA maintenance following a supervised exercise program [24]. To our knowledge, no studies have been performed yet on the acceptability of remote coaching following supervised oncology rehabilitation, using the TFA model.

The first aim of this study was to get insight into perceived determinants of PA maintenance in the transition from a supervised exercise oncology rehabilitation program to habitual PA in the community. The second aim was to assess the acceptability of a 6-month remote coaching intervention to stimulate PA maintenance following a supervised exercise program in cancer survivors.

## Methods

### Study design and theoretical frameworks

A qualitative study design with a phenomenological approach was used and semi-structured interviews were conducted. Procedures of data collection complied with the Declaration of Helsinki and were approved by the Ethical Review Board of MUMC + (registration number 18–050). Results were reported according to the Consolidated criteria for Reporting Qualitative research (COREQ) [25].

The interviews investigated:

1. The perceived determinants of PA maintenance following a supervised oncology exercise program. This part was explorative in nature, using the COM-B model as a theoretical framework. An explorative approach was applied to entangle the complex interaction of factors influencing PA behaviour.
2. The acceptability of a remote coaching intervention in this period. This part was explanatory in nature, using the framework of acceptability (TFA) as a theoretical basis.

### COM-B Model

**Fig. 1:**
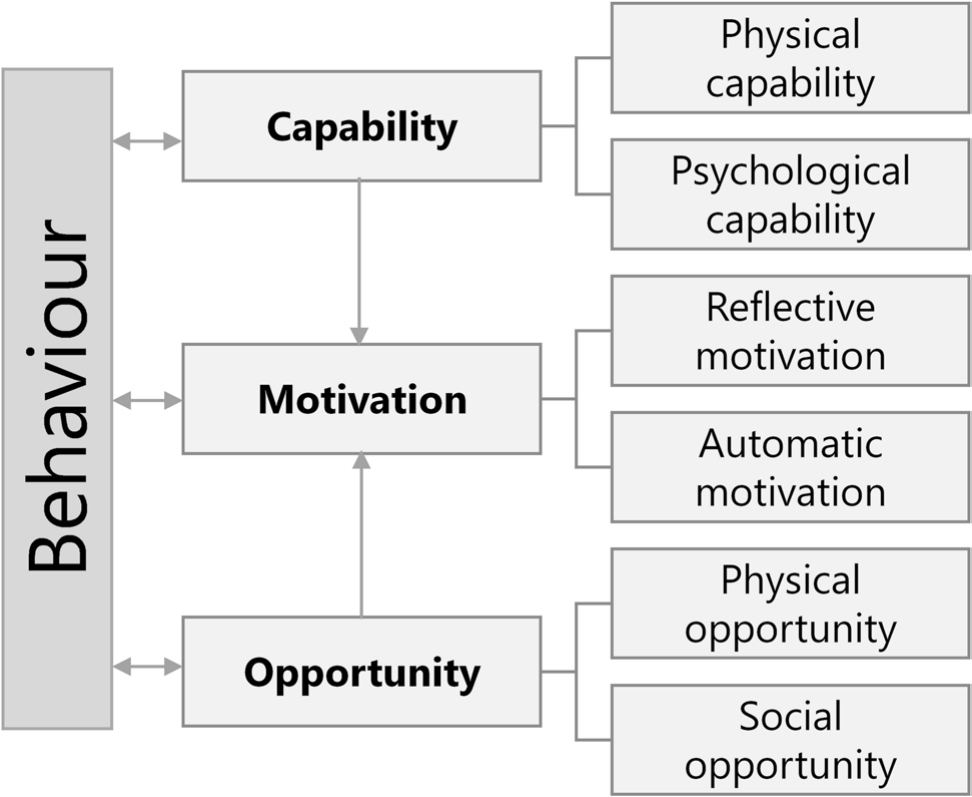
COM-B model. Reproduced from [19]

PA maintenance was explored from the perspective of the COM-B model. In this model, Capability (physical and psychological), Opportunity (social and physical), and Motivation (reflective and automatic) are seen as drivers of behaviour. Motivation is the central mediator of the model which is affected by Capability and Opportunity (Fig. 1). In the COM-B model, behaviour is seen as part of a complex system of interacting factors [19, 20]. The interpretation of the constructs of the COM-B model in the current study is described in Online Resource 1. The analysis of perceived determinants of PA maintenance had an explorative approach because the contribution of the different constructs of the COM-B model to PA maintenance is complex and remains unknown. Focusing only on these distinct constructs might result in a thin description of determinants without getting to the root of the problem of PA maintenance. Therefore, the COM-B constructs were guiding during the interviews and analyses but not restrictively defining, and key themes were allowed to emerge apart from the constructs of the model.

#### TFA

The analysis of intervention acceptability was based on the constructs of the TFA (i.e. affective attitude, self-efficacy, perceived effectiveness, ethicality, intervention coherence, burden, opportunity costs). The interpretation of these constructs as applied to our context is described in Online Resource 1. In the TFA model, all aspects of acceptability are captured in the different constructs [21]. These constructs were used explanatory, meaning that the interviews aimed to gain insight into whether, to what extent, and how each construct contributed to the overall acceptability of the intervention. An explanatory approach was chosen because each of the constructs of the TFA explains an essential part of the acceptability and therefore should be included, but additional overarching themes were allowed to emerge as well.

### Study context

Participants for this study were recruited from a randomized controlled trial (RCT) on the effectiveness of 6-month remote coaching following a 10-week supervised exercise program as part of multidisciplinary oncology rehabilitation at the Maastricht University Medical Center + (MUMC +), Maastricht, the Netherlands. The MUMC + is a university hospital and is recognized as a Comprehensive Cancer Center by the Organization of European Cancer Institutes. The supervised exercise program was part of usual care and the content of this program is described elsewhere [26]. After completion of the supervised program, 97 participants were included in the RCT and randomized to either the intervention group or the control group. The control group (C) received no additional interventions after completing the supervised exercise program. Participants in the intervention group (I) received a remote coaching intervention. Measurements of PA behaviour and physical- and psychosocial functioning were carried out at baseline and after 6 months.

### The remote coaching intervention

The 6-month remote coaching intervention was delivered by a community-based sports organization (Maastricht Sport, Municipality of Maastricht, the Netherlands) and aimed to stimulate patients to increase their PA levels. This intervention was not newly developed but was identified as potentially beneficial for PA maintenance in cancer survivors following a supervised exercise program and is now tested in the evaluation phase of the MRC framework, in the current study and the RCT. Involved coaches had at least a bachelor’s degree in Sport Science or Sports and Movement Education, were trained in behaviour change techniques, and had experience with delivering the intervention. During a face-to-face intake assessment at the Department of Physical Therapy at the Maastricht UMC + , the coach obtained information about the subjects’ personal motivation and PA preferences using the COM-B model. The coaches identified facilitators and barriers for behaviour change in these three constructs and adapted the coaching accordingly. After the intake, the program consisted of individually tailored, remote coaching. The coaching took place via phone calls or e-mails, dependent on personal preferences. In the first 3 months, the coach approached the subjects weekly. Thereafter, the coach evaluated the individual progress, and the frequency was reduced to one contact moment per month.

### Participants

Criterion sampling was used to recruit participants from both the intervention group (I) and the control group (C) of the RCT until data saturation [27] was reached. The eligibility criteria for this study were the same as for the RCT. Patients were eligible to participate in this study when they were ≥ 18 years of age; were suffering from physical, and/or psychosocial complaints and/or chronic fatigue; had completed active medical treatment (i.e. surgery, chemotherapy, radiotherapy, stem cell transplantation) and a 10-week exercise program, as part of multidisciplinary oncology rehabilitation. Patients were excluded if they had insufficient understanding of the Dutch language, were in an unstable phase of disease (e.g. receiving palliative treatment), and scheduled for chemotherapy, radiation, or invasive surgery in the 6 months after completing the exercise program and if they were unable to perform exercise activities without supervision (i.e. because of risk of falling or injuring). They were approached to participate during a phone call for planning their follow-up measurement for the RCT. All participants gave written informed consent.

### Interview procedures

Face-to-face, semi-structured interviews took place at the Department of Physical Therapy at MUMC + . Interviews were planned on the same day as the follow-up measurements for the RCT and took approximately 30 min. To avoid bias, interviews were conducted by an independent researcher (NS) not involved in the rehabilitation program or the RCT. A second independent researcher (LM) was present to take field notes, check for interview completeness, ask additional questions when needed, and give a verbal summary for verification at the end. Participants received a written summary of the interview for a member check. The interview guide was designed by the researchers a priori, based on the COM-B model (exploratory) and TFA (explanatory), and was adapted once, after the seventh interview, to add more in-depth questions to further explore the initial interview guide’s themes (see Online Resource 2). Interviews were recorded using a digital voice recorder and transcribed verbatim afterwards. Recordings were deleted after transcription was completed.

### Coding and analysis

Template analysis [28] was conducted to code the transcripts, using NVivo V.12. Coding was performed by two researchers (NS, AW) who were guided by a third, experienced qualitative researcher (JS). An a priori coding template was developed, based on the COM-B model and the TFA. Subsequent template versions evolved and were allowed to deviate from the initial frameworks, based on emerging topics. After coding the first interview, the template was adapted to an initial template. After each two to three interviews, the coding template was adapted based on emerging topics. Codes were added, removed, or merged as appropriate. The transcripts of the first three interviews were coded independently by two researchers and discussed afterwards until consensus was reached. After the first three interviews, transcripts were coded by one researcher and discussed afterwards with a second researcher for researcher triangulation. After 16 interviews, the fifth and final version of the coding template was formed. During the last three interviews, no new codes emerged for both research aims, which indicated that code saturation was reached [27].

### Researcher characteristics and reflexivity

In this paragraph, the background and characteristics of researchers involved in data collection and analysis are reported, in order to provide insight into possible researcher biases. During the course of the study, AW was working at the Department of Physical Therapy of the MUMC + as an embedded scientist in the field of human movement science. She was working partly as a physical therapist, specialized in exercise oncology rehabilitation and treating patients with neurological disorders. At the same time, she was working as a PhD candidate in the field of oncology rehabilitation. The current study and the aforementioned related RCT were part of her PhD project. Because of her close involvement in this research and the patients in the oncology rehabilitation, she did not conduct the interviews and worked together with independent researchers (NS, LM, and JS) during data analysis in order to minimize the risk of bias. NS got her Bachelor’s degree in physical therapy, was an MSc Human Movement Science student at the time of the study, and was working as a research trainee at the Department of Physical Therapy of the MUMC + . LM was employed as a physical therapist specialized in orthopaedics and geriatrics and PhD candidate in the field of orthopaedics, at the Department of Physical Therapy of the MUMC + . JS was working as an associate professor at the Department of Anatomy and Embryology at the University of Maastricht and is a senior researcher in the field of human movement science, with experience in qualitative research and a focus on physiotherapy. TL was working as a professor of Hospital-based Physiotherapy and has contributed to several qualitative studies in this field. MB was working as a sports physician and senior researcher in the field of oncology. They have both provided supervision during the course of the study and their expertise contributed to triangulation. AW, NS, LM, and MB had less experience with qualitative research but they received the necessary training and worked closely together with JS and TL during the conduct of this study.

## Results

### Participants

Between March and June 2021, twenty-two patients were eligible to participate in this study. Three of them declined because of the required time investment or personal reasons, resulting in a final sample of 19 participants (16 women /3 men). All participants answered questions about determinants for PA maintenance. Twelve participants (63.2%) received the coaching intervention and answered questions about the acceptability of this intervention, additionally. The participant characteristics and group distribution are described in Table 1.

**Table 1:** Participant characteristics

Participant	Group (I/C)	Sex (F/M)	Age category (years)	Cancer diagnosis	Medical treatment	Time since treatment completion* (months)
P01	I	F	46–55	Breast	Surgery; CT	13–15
P02	I	M	56–65	Colorectal	Surgery	16–18
P03	C	F	46–55	Breast	Surgery; RT	13–15
P04	I	F	56–65	Breast	Surgery; CT; RT; HT	13–15
P05	C	F	18–35	Breast	Surgery; CT; RT; IT	16–18
P06	I	F	56–65	Breast	Surgery; RT	16–18
P07	I	F	56–65	Breast	Surgery CT; RT; HT	13–15
P08	C	F	18–35	Breast	Surgery; CT; RT	9–12
P09	I	F	56–65	Breast	Surgery; CT; HT	9–12
P10	C	F	> 65	Lung	CT; RT	13–15
P11	C	F	56–65	Oesophagus	Surgery; CT; RT	13–15
P12	I	M	56–65	Prostate	Surgery; RT	13–15
P13	I	M	35–46	Testis	Surgery; CT	9–12
P14	I	F	18–35	Leukaemia	CT	13–15
P15	I	F	> 65	Breast	Surgery; CT, RT; IT; HT	13–15
P16	C	F	56–65	Breast	Surgery; CT; RT	9–12
P17	I	F	36–45	Melanoma	Surgery	13–15
P18	I	F	> 65	Lymphoma	CT; IT	9–12
P19	C	F	36–45	Breast	Surgery	16–18

^*^Time since active medical treatment, hormone therapy, and immunotherapy not included

*I*, intervention group of the RCT; *C*, control group of the RCT; *F*, female; *M*, male; *CT*, chemotherapy; *RT*, radiotherapy; *HT*, hormone therapy; *IT*, immunotherapy

### Results part I: determinants for PA maintenance

**Fig. 2:**
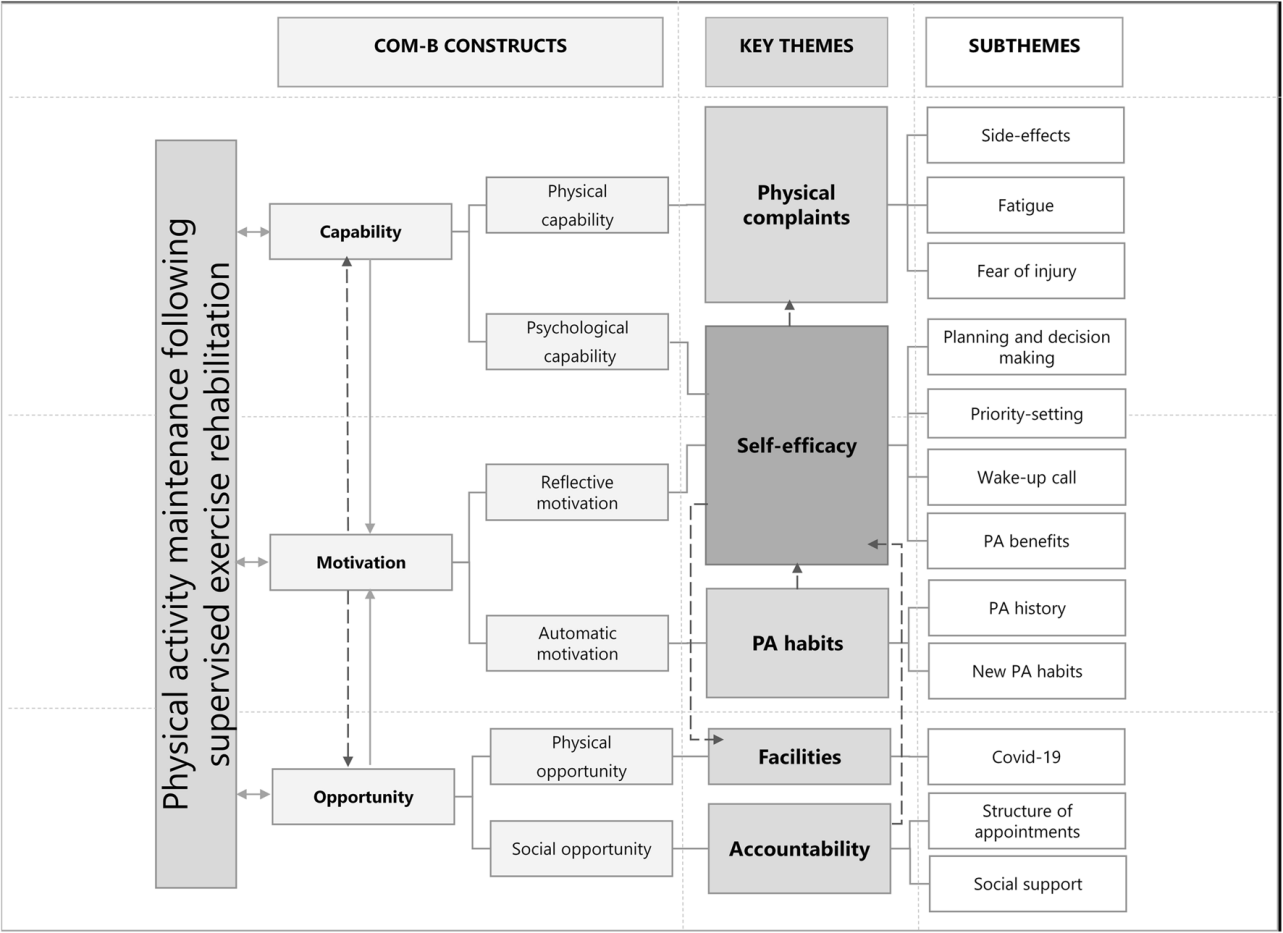
Key determinants and determinants of physical activity maintenance following supervised exercise rehabilitation, clustered in the constructs of the COM-B model. Arrows to show the relation between key themes, with self-efficacy as a central theme

Key themes regarding perceived determinants of PA maintenance were self-efficacy, PA habits, accountability, physical complaints, and facilities. These themes are explained below with quotes and related determinants. In addition, key themes and perceived determinants were clustered according to the constructs of the COM-B model in Fig. 2.

### Self-efficacy

Participants described that confidence to perform PA enabled them to maintain PA levels and overcome perceived barriers. In contrast, feelings of insecurity and incompetence discouraged patients from being active, even when the circumstances were optimal. This kind of behavioural control is often referred to as “self-efficacy”, and this topic came up frequently during the interviews. Self-efficacy is seen as an important part of reflective motivation and can be defined as “people’s belief in their capabilities to take control over their own functioning and over events that affect their lives” [29]. Level of self-efficacy seemed to be related to many other perceived determinants and was therefore an important key theme. Some participants mentioned that they experienced PA as a way to take control of their recovery.

**P08(C)**: “Exercising gives me the feeling that I have influence over my recovery. It’s hard for me to let go of control, and this puts you in control.”

Some participants had intentions to perform PA regularly but did not believe they were capable to sustain it. In these cases, the inability to maintain PA levels seemed to be related to a lack of self-efficacy.

**P01 (I)**: “When you are walking on the treadmill in the hospital you know you have to, so you just do it. Someone is standing next to you and you just keep going, because you know you have to and you feel safe with that person. But at home, you ask yourself ‘Why do I have to do that?’ I am too weak and something might happen or I might fall”.

Self-efficacy was increased by positive beliefs about and experiences with PA. Reflections about potential benefits were enablers for PA maintenance because participants wanted to take control over their own functioning. The belief that PA could improve recovery and general health and reduce the risk for cancer recurrence increased the level of self-efficacy regarding PA maintenance. Participants also believed and experienced that PA leads to improved energy levels and physical and mental state.

**P01(I)**: “I don’t want the cancer to come back. I don’t think it will, but I noticed that I feel better after walking or exercising. When you stay physically active, you get healthier, you can breathe better. And also mentally… It’s something you can do for yourself, for your health”.

For some participants, their cancer diagnosis was a “wake-up call”. It increased their awareness about the benefits of healthy living and the role of PA in this, leading to higher levels of self-efficacy.

**P13(I)**: “It was a wake-up call, my disease. I wanted to take care of myself and wanted to get back on my feet. Well, yes that was actually my biggest motivation to exercise”.

Reaching high levels of self-efficacy for PA maintenance is not only the result of reflective motivation but also requires certain psychological capabilities. This includes understanding the risks of an unhealthy lifestyle, but also capabilities for planning and decision-making. Participants reported that attention and time for PA in daily-life time schedules, but also decisions about priorities in life often changed after the diagnosis of cancer.

**P14(I)**: “Physical activity is important and it’s higher on my priority list now. If it’s necessary I just reschedule work or other things”.

When PA was not considered a priority, this was mostly not literally mentioned during the interviews. However, one participant mentioned that getting fit was not “top of mind” at that moment, because of changes in daily routines and the preference to take it slow during recovery. Perceived determinants related to the self-efficacy theme could be linked to the following constructs of the COM-B model: *reflective motivation* and *psychological capability* (Fig. 2).

### PA habits

During the interviews, participants who successfully maintained PA often shared their experiences with the process of habit-forming. When participants regularly performed PA before their diagnosis, this positively affected PA maintenance because they already had a PA routine before and could pick up their old schedule. These participants with prior PA habits often expressed positive emotions towards PA. Besides, participants who already performed PA in the past seemed to be more confident about their capabilities for PA maintenance, leading to higher levels of self-efficacy.

**P08(C)**: “For me it was not that hard to sustain it, to stay physically active, because I have always been before. Before the diagnosis as well. I just really enjoy hiking”.

Some participants were able to form PA habits during the study period, while they did not perform regular PA before.

**P13(I)**: “I came to the point that my PA behaviour was stable, as a part of my routine. I used to exercise before occasionally. Now it’s more structured and I’m able to sustain it”.

Habits are the result of automatic brain processes and determinants related to habits belong to the construct of *automatic motivation* in the COM-B model (Fig. 2).

### Accountability

Participants mentioned that they needed some kind of structure in order to make PA part of their routine. Scheduled appointments with others were seen as a source of structure and were perceived to increase accountability for PA maintenance. Accountability can be defined as “the fact of being responsible for your decisions or actions and being expected to explain them when you are asked” [30]. Accountability was a key theme during the interviews and was discussed from several perspectives.

Participants reported that they felt accountable to show up when they had an appointment with their physical therapist, sports instructor, or peers. For some participants who received coaching, the expectation of the next phone consultations made them feel accountable for PA maintenance. When a phone consultation was scheduled with the coach, they knew they would be asked to report on their PA behaviour, and they felt accountable to perform PA. In this way, accountability did also increase the level of self-efficacy, because the fact that participants had an appointment they had to meet, or an expectation to fulfil, made them feel more confident about being able to stick to their PA plans.

**P04 (I)**: “I liked that I received a phone call once a week, which gave me a feeling of accountability. It is a good thing to be more or less accountable for physical activity, because you know you have to report it to the coach. It’s the same with an appointment to participate in a group-based exercise activity, which I believe you would only cancel if you have a good reason for it. Therefore, you’re more likely to participate in that activity”.

**P03(C)**: “Unfortunately, I have little self-discipline to start exercising. I know that it’s good for me, but it just works better if someone tells me ‘You have to be there at a certain time’ or ‘Why did you not show up last week?’. I just need that kind of structure to feel accountable”.

Accountability relies on an expected social interaction and is therefore closely related to social support [31]. Respondents mentioned that social support motivated them for PA maintenance. Sometimes, this support was offered by relatives who actually exercised together with the patients, but other forms of support were mentioned as well, like social support from the coach, peer support, playing with grandchildren, or walking the dog. Participants felt accountable to relatives who supported their PA behaviour because they wanted to fulfil their expectations.

**P04(I)**: “The grandchildren told me ‘Grandma, you have to exercise!’ My granddaughter told me: ‘Come on, grandma, let’s go’. That stimulated me. Or my grandson who said: ‘Grandma, you have to lift me, that will make you strong’”.

Social support did not only offer structure through accountability, but it made PA also more fun and enjoying, resulting in positive emotions towards PA.

**P09(I)**: “With support, or a sports club or something it’s easier for me. I find it more enjoying and fun and I’m better able to keep up with it”.

Determinants related to accountability can be linked to the construct *social opportunity* in the COM-B model (Fig. 2).

### Physical complaints

The influence of physical complaints on PA maintenance was discussed. Even after completing the supervised exercise program, participants were often confronted with physical complaints. Chronic fatigue and treatment side effects were often mentioned during the interviews. When patients experienced these kinds of physical disabilities, this was a barrier for PA maintenance.

**P01(I)**: “The hormone therapy was really bad for my body. The side-effects almost turned me disabled. I had difficulties with standing up, with walking. Therefore, the doctor and I decided to stop the hormone therapy and that was a very positive experience. I felt much better and was able to walk and cycle!”.

Some patients mentioned physical complaints but described how they maintained PA despite of this. The ability to cope with physical complaints seems to depend on the level of self-efficacy. Some patients were capable to maintain PA when experiencing physical complaints or were able to pick up PA habits after recovering, while others could not. Some participants felt like they were caught in a “vicious circle”. They felt incapable to perform PA because of physical complaints and consequently felt less fit, more fatigued, or even depressed as a result of being inactive.

Not only the presence of but also the fear of developing physical complaints was a barrier for PA. Some participants had a fear of injury when exercising independently. The confidence to perform PA independently was related to the patient’s level of efficacy.

**P12(C):** “If you do it all by yourself, the chance of getting injured is very high. You have to perform the right exercises”.

Physical complaints can be linked to the construct of *Physical Capability* in the COM-B model (Fig. 2).

### Facilities

The accessibility of sports and rehabilitation facilities and thereby PA maintenance were negatively affected by the COVID-19 pandemic and related measures. This topic often emerged during the interviews. Some respondents reported that they managed to maintain PA levels until the COVID-19 pandemic commenced, but failed to continue when facilities had to close. The ability to adapt their PA routine in this situation differed between respondents and was related to the level of self-efficacy.

**P04(I)**: “Covid-19 was a disadvantage, has made things hard, because going to exercise independently (in community-based facilities, which had to close) was just not possible”.

The theme of facilities is related to the constructs of *physical opportunity* in the COM-B model. (Fig 2)

### Results part II: acceptability of the coaching intervention

The seven constructs of the TFA were discussed with participants who received the coaching intervention (*N* = 12), to get insight into whether, to what extent, and how each of the constructs contributed to the overall acceptability of remote coaching. In addition, three TFA overarching key themes were determined. Overlap was seen with the key themes for determinants of PA maintenance in part I. The first key theme is accountability since the remote coaching intervention offered structure and social support and thereby led to an increased feeling of accountability. This influenced the affective attitude as well as the perceived effectiveness of participants towards the intervention. The level of self-efficacy was the second key theme for the acceptability because respondents’ belief in their capabilities to follow the advice of the coaching determined the perceived effectiveness. Besides, participants mentioned that the coaching made them feel confident, which could lead to an increase in the level of self-efficacy. The third overarching theme was the remote nature of the intervention, which was convenient for some participants, but not for others. The participants’ experience with the remote nature of the intervention influenced their affective attitude, perceived effectiveness, self-efficacy, and the burden.

### Affective attitude

Overall, participants had positive experiences with the coaching intervention. They appreciated the personal contact, the attention, and kindness of the coaches.

**P18**: “It is about the attention. She was asking me how it went and I told her what PA activities I did that week and that was nice”.

Participants also described that the remote coaching intervention made them feel accountable for performing PA.

**P02**: “It is nice to have an appointment that makes you feel accountable to perform physical activity like you intended. To report how it went and to be more or less accountable”.

However, some participants would have preferred a coaching intervention with physical appointments. They felt a phone call was not enough to motivate them. It should be noted that for some of the participants, even the first appointment, which is usually a physical appointment, had been via a phone call, due to COVID-19 restrictions.

**P12:** “If they really have to stimulate me to perform PA, because I can’t do it, or because I’m not motivated, then a phone call is not enough. Then you really need to see someone face-to-face”.

### Self-efficacy

As already described in the first part of this article, self-efficacy is a key theme in PA maintenance. In the TFA, self-efficacy refers to persons’ confidence about their ability to perform the required behaviour. Most participants described that they felt confident to follow the coaches’ advice. They mentioned that PA advices were personalized and based on shared decision-making. However, for some participants, it was difficult to stay active, despite the advice from the coach, indicating low levels of self-efficacy.

**P04**: “I feel bad about myself, that I’m not capable to do it all by myself. I just can’t do it. She called me and asked ‘is there anything I can do for you?’ But in the end, I have to do it by myself, right?”.

### Perceived effectiveness

The majority of participants believed the coaching intervention was effective for improving PA maintenance. They mentioned that the coaching stimulated them to maintain PA, by offering a source of structure, accountability, social support, and confidence after the supervised exercise program.

**P07**: “Without the motivational coaching intervention I would not have exercised, I am 100% sure about that. Maybe I would have performed an online program for three or four weeks, but then I would have stopped. The coaching really offered me a structure to keep exercising”.

However, a few participants perceived no effect, because they had the feeling they had to perform PA by themselves and the advice did not help them with this, or because they already felt capable to perform PA independently without coaching.

**P12**: “I believe the coaching is effective for people who need it, but for me it was just a pleasant short chat. I could not say that it helped me”.

#### Ethicality

Expectations about the coaching intervention were diverse. Some of the participants well-understood the content of the intervention beforehand because they read about it in the research participant information. Others did not know what to expect or expected exercise training given by a sports coach instead of remote coaching.

**P13**: “Actually I didn’t know exactly what it would entail, the coaching. But they already told me that it was not someone who sets up a training program for you, it’s more like a source of accountability, someone who contacts you”.

Participants mentioned that the added value of the coaching intervention might differ between individuals, depending on their personal needs. They believed that especially persons who have difficulties with maintaining PA, might benefit from the coaching.

**P17:** “For people who have difficulties with exercising, or who don’t regularly perform exercise, I think it might increase accountability and give extra motivation to push through. I think it depends on the person”.

### Intervention coherence

Participants well-understood the aim of the coaching intervention and were able to describe this. Stimulating PA maintenance and motivation were most often mentioned as the main goal. Participants also related the aim of this intervention to health improvement, showing they were aware of PA benefits.

**P17**: “To motivate people for physical activity and to sustain it. And to actually become aware of the importance. We all know that physical activity is important and healthy, but you have to keep doing it”.

While the goal of the intervention was clear, some participants questioned whether remote coaching was the most appropriate mode of delivery. They believed that physical appointments were needed to stimulate PA maintenance.

### Burden

The majority of participants did not experience the coaching intervention as a burden. They thought it was convenient to receive the coaching by phone and mentioned that the planning by the coaches was flexible. Two participants experienced the calls as a burden sometimes, when the coaches called while they were busy.

**P13**: “It was no burden because the coach was very flexible. We had an appointment at a certain time, but when that turned out to be inconvenient she called half an hour later”.

The remote nature of the intervention positively affected the acceptability of the intervention in some participants and negatively affected it for others.

### Opportunity costs

No opportunity costs were mentioned during the interviews.

## Discussion

The aim of this qualitative study was twofold. First, we wanted to explore determinants of PA maintenance during the transition from supervised exercise oncology rehabilitation to habitual PA in the community. Second, we wanted to investigate whether and for what reasons a remote coaching intervention was perceived acceptable by cancer survivors during this period.

Determinants for PA maintenance were explored, and five key themes were identified, covering and linking all constructs of the COM-B model. The Capability of participants to maintain PA was dependent on physical complaints (physical capability) and on the level of self-efficacy needed for tasks like planning and priority-setting (psychological capability). Self-efficacy was not only dependent on patients’ capability but also related to their motivation for optimizing health and recovery (reflective motivation). Besides, motivation for PA maintenance relied on automatic habitual processes, and patients with prior PA habits are more likely to successfully maintain PA (automatic motivation). The possibility of participants to maintain PA was dependent on the accessibility of facilities, which was negatively affected by the COVID-19 pandemic (physical opportunity), and on their accountability for PA maintenance, which was reinforced by social support (social opportunity). The fact that physical complaints like chronic fatigue and treatment side effects emerged as a perceived barrier for physical activity after supervised rehabilitation following medical treatment implies that more support is needed to achieve long-term PA. Reassurance and encouragement by healthcare providers, including the physician, are required for patients to be able to overcome these barriers. Patients should be informed that performing PA is safe for them and even beneficial. Moreover, the fact that cancer survivors still experience side-effects long after completion of the treatment advocates the integration of survivorship care earlier in the patient journey to prevent for side effects.

According to the COM-B model, Motivation is the central mediator of behaviour, which is affected by Capability and Opportunity. However, we believe that Motivation conversely affected the Capability and Opportunity to maintain PA as well. Patients with higher levels of self-efficacy were more likely to overcome barriers in the construct of Capability and Opportunity, like the burden of and fear of physical complaints and the limited accessibility of facilities during the COVID-19 pandemic. Patients who had high levels of self-efficacy believed in their capabilities to perform PA despite these barriers and were able to overcome them, while patients who did not, mentioned that they could not perform PA because of these barriers. Therefore, arrows were added in Fig. 2, pointing back from Motivation to Capability and Opportunity. These findings confirm the statement of the COM-B model that behaviour is a complex process which is partly an entangled system of interacting factors [19, 20].

Remote coaching was perceived as generally acceptable to cancer survivors who completed a supervised exercise program. Key themes for acceptability were self-efficacy, accountability, and the remote nature of the intervention. Participants reported that the coaching had positive effects on PA maintenance, by offering structure and confidence and consequently improving accountability and the level of self-efficacy. The perceived effectiveness was also dependent on the level of self-efficacy. This implies that it could be useful to assess the level of self-efficacy at the start of a remote coaching intervention and adapt the coaching accordingly. The remote nature of the intervention positively affected the acceptability in some participants and negatively affected it in others. Some participants would have preferred face-to-face appointments instead of or in addition to phone calls, while others found the remote nature convenient.

Our findings about determinants for PA maintenance were broadly in line with those of previous studies. Gell et al. explored female cancer survivors’ perspectives on remote coaching interventions to improve PA maintenance and identified five themes with great similarities to our study findings: accountability to a remote partner; plan Bs planning for barriers; the habit cycle; convenience through technology; and reclaiming health ownership [23]. Ferri et al. reported that the transition from a supervised environment to everyday life was a significant barrier to maintain exercise participation following a hospital-based exercise program. Participants had concerns about fitting exercise in daily life, particularly because participants would return to work [11]. This concern did not emerge during the interviews of the current study, which could be explained by the fact that some participants in our study did not return to work yet in the 6 months after completing the exercise program, or were retired. Although not specifically related to work, difficulties with fitting PA into everyday life were mentioned during the current study as well. Cantwell et al. conducted an exploratory, qualitative study of the experiences of patients across the cancer journey [32]. They reported that regular PA provided a “vehicle for recovery” and created a sense of “self-power”, which is in line with our findings about self-efficacy. Environmental, patient-related, and treatment-related barriers were reported as well and were similar to our findings. In contrast to our study, financial costs were a perceived barrier for PA participation. In the current study, the financial burden was discussed in some interviews but was never a reason to quit PA participation. This may be due to the fact that options for insurance-covered, low-cost, or even free PA activities were discussed with the participants at the end of the exercise program. These findings emphasize the importance of the availability of PA-promoting interventions for all cancer survivors, regardless of their financial status. Telehealth interventions have the potential to reach many patients, requiring fewer resources than face-to-face interventions [14.] The current and previous findings fit within the Cancer Rehabilitation to Recreation (CaReR) Framework. In the CaReR Framework, a stepped-care approach is proposed, considering the importance of behaviour change and routine assessment. In accordance with our findings, the framework emphasized the importance of self-efficacy, by recognizing that the most suitable settings of PA interventions vary depending on the level of self-efficacy and recognizing that PA counselling should be offered throughout the patient journey to build self-efficacy [33].

Our findings about the acceptability of remote coaching had similarities with the findings of a review about the use of telehealth in cancer survivors. In this review, high satisfaction with remote interventions was reported, but the importance of customization was emphasized. The preference for in-person follow-up was reported, like in our study, and visual elements were appreciated when interventions were remote [34]. These findings indicate that a combination of physical and remote appointments could be a future solution and video calls could potentially add benefit compared to normal phone calls. Gell et al. describe that remote interventions are acceptable to support PA in female cancer survivors, when known preferences are incorporated, to focus on personal intentions and goals [24]. In the current study, personal preferences were mapped during the intake appointment using the COM-B model and incorporated into the remote coaching intervention. Although the coaching intervention was generally considered acceptable, participants mentioned that the added value of the intervention depended on personal needs. During this study, all participants in the intervention group of the RCT received the coaching intervention. However, in daily practice, remote coaching following a supervised exercise program should be offered only when patients need it, are open to it, and if the intervention matches or can be tuned to their personal needs and preferences. Triage would be required to determine if there is an indication for PA maintenance interventions and which intervention is most suitable. The optimal mode of delivery, content, duration, and intensity of the coaching might depend on personal factors, like the patient’s social environment and their level of self-efficacy, and should be personalized. The integration of triage and stratification could be based on comparable interventions, which are already successfully implemented, like the Coach2Move approach [35]. This is a personalized and goal-oriented physical therapy intervention aimed at improving long-term levels of physical activity in which patients are stratified to one of three intervention profiles with a pre-defined number of sessions. The intervention is provided face to face, and future research should explore the potential of combining physical and remote appointments. Foster et al. identified determinants of PA maintenance in patients with gastrointestinal cancer and reported that patients will likely need minimal support for PA maintenance when they perform a PA activity they enjoy. Besides, they describe that participants who have a history of exercising, hold strong values of PA importance, which is in line with our findings about PA habits [36].

A strength of this study was the fact that perceived determinants for PA maintenance after a supervised exercise program were assessed in both cancer survivors that did and did not receive a follow-up intervention. In this way, we tried to get insight into patients’ experiences with this transition phase from multiple perspectives. Furthermore, the acceptability of a potential intervention for PA maintenance was investigated, which is important for optimizing the intervention and successful implementation in daily practice. A novel aspect of this study can be found in the contribution of telehealth to promote PA maintenance in cancer survivors. The fact that we included mainly women with breast cancer can be seen as a study limitation. Besides, all participants took part in an RCT to the effectiveness of remote coaching following supervised exercise rehabilitation. Participants who were included in this RCT were potentially more motivated for PA, and their behaviour and opinions may have been influenced by the research information. Because of these reasons, the findings of this study might not be transferable to all cancer survivors. Finally, this study was conducted during the COVID-19 pandemic. Findings about determinants for PA maintenance and acceptability of remote coaching would probably have been different if not examined during the COVID-19 pandemic.

Future studies should focus on identifying cancer survivors at risk for turning inactive following structured exercise programs and designing appropriate follow-up interventions for patients with different needs. Since it could be challenging to reach and motivate these patients to participate in these interventions, appropriate methods to achieve this should be investigated as well. Besides, future research should focus on further evaluation, refinement, and implementation of the remote coaching intervention. We would propose to use the MRC framework for complex interventions to guide these future steps [22].

In conclusion, the findings of the current study implicate that the transition from supervised rehabilitation to daily life PA is influenced by a variety of determinants that are related to the Capability, Opportunity, and Motivation of the patient. The level of self-efficacy plays a major role in the ability to maintain PA following supervised rehabilitation. Besides, the formation of PA habits, the feeling of accountability, the presence of and fear of physical complaints, and the accessibility of facilities were reported. A remote coaching intervention to promote PA maintenance was perceived acceptable to cancer survivors who participated in a supervised exercise program but could be improved by adding face-to-face appointments. Participants experienced the remote coaching intervention as a source of structure, accountability, social support, and self-efficacy, but the perceived added value of the intervention differed between participants. We believe that interventions for PA maintenance need a personalized approach and should focus on habit-forming and improving self-efficacy, helping patients to overcome PA barriers like work schedules, treatment-related side effects, and adapting during crises like a pandemic.

## Supplementary Information

Below is the link to the electronic supplementary material.

Supplementary file1 (DOCX 16 KB)

Supplementary file2 (DOCX 16 KB)

## Data Availability

Data are available upon reasonable request.

## References

[1] Ebede CC, Jang Y, Escalante CP. Cancer-related fatigue in cancer survivorship. Med Clin North Am. 2017;101(6):1085–97. 10.1016/j.mcna.2017.06.007. 28992856 10.1016/j.mcna.2017.06.007

[2] Jean CY, Syrjala KL. Anxiety and depression in cancer survivors. Med Clinics. 2017;101(6):1099–113. 10.1016/j.mcna.2017.06.005. 10.1016/j.mcna.2017.06.005 PMC5915316 28992857

[3] Jones LW, Courneya KS, Mackey JR, Muss HB, Pituskin EN, Scott JM, et al. Cardiopulmonary function and age-related decline across the breast cancer survivorship continuum. J Clin Oncol. 2012;30(20):2530–7. 10.1200/JCO.2011.39.9014. 22614980 10.1200/JCO.2011.39.9014 PMC3397786

[4] Marques VA, Ferreira-Junior JB, Lemos TV, Moraes RF, Junior JRS, Alves RR, et al. Effects of chemotherapy treatment on muscle strength, quality of life, fatigue, and anxiety in women with breast cancer. Int J Environ Res Public Health. 2020;17(19):7289. 10.3390/ijerph17197289. 33036182 10.3390/ijerph17197289 PMC7579368

[5] Nayak MG, George A, Vidyasagar MS, Mathew S, Nayak S, Nayak BS, et al. Quality of life among cancer patients. Indian J Palliat Care. 2017;23(4):445–50. 10.4103/IJPC.IJPC_82_17. 29123353 10.4103/IJPC.IJPC_82_17 PMC5661349

[6] Buffart LM, Kalter J, Sweegers MG, Courneya KS, Newton RU, Aaronson NK, et al. Effects and moderators of exercise on quality of life and physical function in patients with cancer: an individual patient data meta-analysis of 34 RCTs. Cancer Treat Rev. 2017;52:91–104. 10.1016/j.ctrv.2016.11.010. 28006694 10.1016/j.ctrv.2016.11.010

[7] Kessels E, Husson O, van der Feltz-Cornelis CM. The effect of exercise on cancer-related fatigue in cancer survivors: a systematic review and meta-analysis. Neuropsychiatr Dis Treat. 2018;14:479–94. 10.2147/NDT.S150464. 29445285 10.2147/NDT.S150464 PMC5810532

[8] Friedenreich CM, Stone CR, Cheung WY, Hayes SC. Physical activity and mortality in cancer survivors: a systematic review and meta-analysis. JNCI Cancer Spectr. 2020;4(1):pkz080. 10.1093/jncics/pkz080. 32337494 10.1093/jncics/pkz080 PMC7050161

[9] Sweegers, M.G., Boyle, T., Vallance, J.K. et al. (2019) Which cancer survivors are at risk for a physically inactive and sedentary lifestyle? Results from pooled accelerometer data of 1447 cancer survivors. Int J Behav Nutr Phys Act 16(66). 10.1186/s12966-019-0820-7 10.1186/s12966-019-0820-7 PMC6698042 31420000

[10] Cheifetz O, Dorsay JP, MacDermid JC (2015) Exercise facilitators and barriers following participation in a community-based exercise and education program for cancer survivors. J Exerc Rehabil 11(1):20–9. 10.12965/jer.150183 10.12965/jer.150183 PMC4378345 25830140

[11] Ferri A, Gane EM, Smith MD, Pinkham EP, Gomersall SR, Johnston V. Experiences of people with cancer who have participated in a hospital-based exercise program: a qualitative study. Support Care Cancer. 2021;29(3):1575–83. 10.1007/s00520-020-05647-y. 32740895 10.1007/s00520-020-05647-y

[12] Spark LC, Reeves MM, Fjeldsoe BS, Eakin EG. Physical activity and/or dietary interventions in breast cancer survivors: a systematic review of the maintenance of outcomes. J Cancer Surviv. 2013;7(1):74–82. 10.1007/s11764-012-0246-6. 23179496 10.1007/s11764-012-0246-6

[13] Schmidt MLK, Ostergren P, Cormie P, Ragle AM, Sonksen J, Midtgaard J. “Kicked out into the real world”: prostate cancer patients’ experiences with transitioning from hospital-based supervised exercise to unsupervised exercise in the community. Support Care Cancer. 2019;27(1):199–208. 10.1007/s00520-018-4306-y. 29931489 10.1007/s00520-018-4306-y

[14] Goode AD, Lawler SP, Brakenridge CL, Reeves MM, Eakin EG. Telephone, print, and web-based interventions for physical activity, diet, and weight control among cancer survivors: a systematic review. J Cancer Surviv. 2015;9(4):660–82. 10.1007/s11764-015-0442-2. 25757733 10.1007/s11764-015-0442-2

[15] Gell NM, Grover KW, Savard L, Dittus K. Outcomes of a text message, Fitbit, and coaching intervention on physical activity maintenance among cancer survivors: a randomized control pilot trial. J Cancer Surviv. 2020;14(1):80–8. 10.1007/s11764-019-00831-4. 31776849 10.1007/s11764-019-00831-4

[16] Gomersall SR, Skinner TL, Winkler E, Healy GN, Eakin E, Fjeldsoe B. Feasibility, acceptability and efficacy of a text message-enhanced clinical exercise rehabilitation intervention for increasing ‘whole-of-day’ activity in people living with and beyond cancer. BMC Public Health. 2019;19(Suppl 2):542. 10.1186/s12889-019-6767-4. 31159752 10.1186/s12889-019-6767-4 PMC6546618

[17] Groen WG, van Harten WH, Vallance JK. Systematic review and meta-analysis of distance-based physical activity interventions for cancer survivors (2013–2018): we still haven’t found what we’re looking for. Cancer Treat Rev. 2018;69:188–203. 10.1016/j.ctrv.2018.07.012. 30077954 10.1016/j.ctrv.2018.07.012

[18] Davis R, Campbell R, Hildon Z, Hobbs L, Michie S. Theories of behaviour and behaviour change across the social and behavioural sciences: a scoping review. Health Psychol Rev. 2015;9(3):323–44. 10.1080/17437199.2014.941722. 25104107 10.1080/17437199.2014.941722 PMC4566873

[19] Michie S, van Stralen MM, West R. The behaviour change wheel: a new method for characterising and designing behaviour change interventions. Implement Sci. 2011;6:42. 10.1186/1748-5908-6-42. 21513547 10.1186/1748-5908-6-42 PMC3096582

[20] West R, Michie S (2020) A brief introduction to the COM-B model of behaviour and the PRIME theory of motivation [v1]. Qeios. 10.32388/WW04E6.2

[21] Sekhon M, Cartwright M, Francis JJ. Acceptability of healthcare interventions: an overview of reviews and development of a theoretical framework. BMC Health Serv Res. 2017;17(1):88. 10.1186/s12913-017-2031-8. 28126032 10.1186/s12913-017-2031-8 PMC5267473

[22] Skivington K, Matthews L, Simpson SA, Craig P, Baird J, Blazeby JM, et al. A new framework for developing and evaluating complex interventions: update of Medical Research Council guidance. BMJ. 2021;374:n2061. 10.1136/bmj.n2061. 34593508 10.1136/bmj.n2061 PMC8482308

[23] Dennett A, Harding KE, Reimert J, Morris R, Parente P, Taylor NF. Telerehabilitation’s safety, feasibility, and exercise uptake in cancer survivors: process evaluation. JMIR Cancer. 2021;7(4):e33130. 10.2196/33130. 34854817 10.2196/33130 PMC8768007

[24] Gell NM, Tursi A, Grover KW, Dittus K. Female cancer survivor perspectives on remote intervention components to support physical activity maintenance. Support Care Cancer. 2020;28(5):2185–94. 10.1007/s00520-019-05038-y. 31422476 10.1007/s00520-019-05038-y

[25] Tong A, Sainsbury P, Craig J. Consolidated criteria for reporting qualitative research (COREQ): a 32-item checklist for interviews and focus groups. Int J Qual Health Care. 2007;19(6):349–57. 10.1093/intqhc/mzm042. 17872937 10.1093/intqhc/mzm042

[26] Weemaes ATR, Weijenberg MP, Lenssen AF, Beelen M. Exercise training as part of multidisciplinary rehabilitation in cancer survivors: an observational study on changes in physical performance and patient-reported outcomes. Support Care Cancer. 2022;30(11):9255–66. 10.1007/s00520-022-07351-5. 36066627 10.1007/s00520-022-07351-5 PMC9444699

[27] Saunders B, Sim J, Kingstone T, Baker S, Waterfield J, Bartlam B, et al. Saturation in qualitative research: exploring its conceptualization and operationalization. Qual Quant. 2018;52(4):1893–907. 10.1007/s11135-017-0574-8. 29937585 10.1007/s11135-017-0574-8 PMC5993836

[28] King N, Symon G, Cassell C. Qualitative methods and analysis in organizational research. London: Sage; 1998. 10.1002/9780470696378.ch8.

[29] Bandura A, Freeman WH, Lightsey R. Self-efficacy: the exercise of control. Springer. 1999. 10.1891/0889-8391.13.2.158.

[30] Hornby AS, Cowie AP, Lewis JW. Oxford advanced learner’s dictionary of current English. 3rd ed. Oxford University Press, 1974.

[31] Oussedik E, Foy CG, Masicampo EJ, Kammrath LK, Anderson RE, Feldman SR. Accountability: a missing construct in models of adherence behaviour and in clinical practice. Patient Prefer Adherence. 2017;11:1285–94. 10.2147/PPA.S135895. 28794618 10.2147/PPA.S135895 PMC5536091

[32] Cantwell M, Walsh D, Furlong B, Loughney L, McCaffrey N, Moyna N, et al. Physical activity across the cancer journey: experiences and recommendations from people living with and beyond cancer. Phys Ther. 2020;100(3):575–85. 10.1093/ptj/pzz136. 31588506 10.1093/ptj/pzz136

[33] Dennett AM, Peiris CL, Shields N, Taylor NF. From cancer rehabilitation to recreation: a coordinated approach to increasing physical activity. Phys Ther. 2020;100(11):2049–59. 10.1093/ptj/pzaa135. 32737975 10.1093/ptj/pzaa135

[34] Irurita-Morales P, et al. Use of telehealth among cancer survivors: a scoping review. Telemed e-Health. 2022. 10.1089/tmj.2022.0351. 10.1089/tmj.2022.0351 36445755

[35] Heij W, et al. (2022) Implementing a personalized physical therapy approach (Coach2Move) is effective in increasing physical activity and improving functional mobility in older adults: a cluster-randomized, stepped wedge trial, physical therapy. 102(12). 10.1093/ptj/pzac138 10.1093/ptj/pzac138 PMC10071485 36200397

[36] Foster C, Survivorship M (2020) A typology of physical activity maintenance in people living with and beyond cancer. 10.1136/bmjopen-2020-037136 10.1136/bmjopen-2020-037136 PMC7597473 33122311

