# Supplementary material for: Determinants of physical activity maintenance and the acceptability of a remote coaching intervention following supervised exercise oncology rehabilitation: a qualitative study
Source: J Cancer Surviv. 2023 Sep 21;19(1):149–61. doi: 10.1007/s11764-023-01455-5 (PMC11813816; doi:10.1007/s11764-023-01455-5)
Supplement: Supplementary file 1 — Supplementary file1 (DOCX 16 KB) [file 11764_2023_1455_MOESM1_ESM.docx]

**ONLINE RESOURCE 1: INTERPRETATION OF THEORETHICAL FRAMEWORKS**

**Determinants of physical activity maintenance and the acceptability of a remote coaching intervention following supervised exercise oncology rehabilitation: a qualitative study**, Journal of Cancer Survivorship, Anouk T.R. Weemaes, PT, MSc^1,2^, Judith M. Sieben, PhD, Milou Beelen, MD, PhD, Loes T.M.A. Mulder, PT, MSc, Antoine F. Lenssen, PT, PhD
^1^ Department of Physical Therapy, Maastricht University Medical Center+, Maastricht, the Netherlands
^2^ Care and Public Health Research Institute (CAPHRI), Faculty of Health Medicine and Life Sciences, Maastricht University, Maastricht, the Netherlands, [anouk.weemaes@mumc.nl](mailto:anouk.weemaes@mumc.nl)

**COM-B Model and interpretation.** Reproduced from [1,2]

| Construct | Interpretation |
| --- | --- |
| Capability | Psychological and physical capacity for PA maintenance. |
| Physical capability | Physique and skills required for PA maintenance. |
| Psychological capability | Capacity to engage in the necessary thought processes, comprehension and reasoning for PA maintenance. |
| Opportunity | All the external factors that lie outside an individual that make PA maintenance possible or prompt it. |
| Physical opportunity | Inanimate parts of the environmental system and time affecting the opportunity for PA maintenance. |
| Social opportunity | Other people and organisations affecting the opportunity for PA maintenance. (e.g. culture and social norms) |
| Motivation | All the brain processes that energise and direct PA maintenance |
| Reflective motivation | Analytical decisions, evaluations and plans, conscious intent for PA maintenance (e.g. planning and evaluation) |
| Automatic motivation | Habitual processes, emotional responding to PA maintenance |

**Theoretical framework of acceptability themes and interpretation. Reproduced from [23]**

| Theme | Interpretation |
| --- | --- |
| Affective attitude | How participants feel about the remote coaching intervention |
| Self-efficacy | The participants’ confidence in their ability to follow the advices given during the remote coaching intervention |
| Perceived effectiveness | The extent to which participants perceive the coaching intervention to be effective (to promote PA maintenance) |
| Ethicality | The extent to which the remote coaching intervention had good fit with the participants’ value system and expectations of it |
| Intervention coherence | The extent to which the participants understand the aim of the remote coaching intervention |
| Burden | The perceived amount of effort that was required / the burden to participate in the remote coaching intervention |
| Opportunity costs | The extent to which benefits, profits or values must be given up to engage in the remote coaching intervention |

**REFERENCES**

1. Michie S, van Stralen MM, West R. The behaviour change wheel: a new method for characterising and designing behaviour change interventions. Implement Sci 2011;6:42 <https://doi.org/10.1186/1748-5908-6-42>.
2. West R, Michie S. A brief introduction to the COM-B Model of behaviour and the PRIME Theory of motivation [v1]. Qeios 2020. <http://doi.org/10.32388/WW04E6.2>
3. Sekhon M, Cartwright M, Francis JJ. Acceptability of healthcare interventions: an overview of reviews and development of a theoretical framework. BMC Health Serv Res 2017;17(1):88 <https://doi.org/10.1186/s12913-017-2031-8>
