# Supplementary material for: Determinants of physical activity maintenance and the acceptability of a remote coaching intervention following supervised exercise oncology rehabilitation: a qualitative study
Source: J Cancer Surviv. 2023 Sep 21;19(1):149–61. doi: 10.1007/s11764-023-01455-5 (PMC11813816; doi:10.1007/s11764-023-01455-5)
Supplement: Supplementary file 2 — Supplementary file2 (DOCX 16 KB) [file 11764_2023_1455_MOESM2_ESM.docx]

**ONLINE RESOURCE 2: INTERVIEWGUIDE**

**Determinants of physical activity maintenance and the acceptability of a remote coaching intervention following supervised exercise oncology rehabilitation: a qualitative study**, Journal of Cancer Survivorship, Anouk T.R. Weemaes, PT, MSc^1,2^, Judith M. Sieben, PhD, Milou Beelen, MD, PhD, Loes T.M.A. Mulder, PT, MSc, Antoine F. Lenssen, PT, PhD
^1^ Department of Physical Therapy, Maastricht University Medical Center+, Maastricht, the Netherlands
^2^ Care and Public Health Research Institute (CAPHRI), Faculty of Health Medicine and Life Sciences, Maastricht University, Maastricht, the Netherlands, [anouk.weemaes@mumc.nl](mailto:anouk.weemaes@mumc.nl)

- Question. Did you receive the remote coaching intervention or not
  (intervention/control group)?

**Part I. Determinants of PA maintenance (all participants)**

- Question. What were your experiences with the transition phase from a supervised rehabilitation program to habitual PA in the community?
- Question. How is exercise/PA currently going?

Capability

- Question. To what extent were you able to maintain PA levels and/or exercise independently beyond completion of the supervised rehabilitation program?
- Question. What did you learn during the supervised rehabilitation program (and the remote coaching?)

Opportunity

- Question. In what way did your current living- and social situation influence your PA maintenance?
- Question. Which environmental factors made it easier/more difficult for you to perform PA? What did you perceive as barriers and enablers for PA maintenance?
- Question. What role did (lack of) social support play in PA maintenance? And professional guidance?

Motivation

- Question. To what extent were you motivated for PA maintenance in the past six months? Did this change over time and what motivated you?
- Question. Did you feel confident to perform PA independently?
- Question. Did you perform PA regularly in the past, before you were diagnosed with cancer?
- Question. What are your beliefs about PA benefits?

**Part II. Acceptability of remote coaching (participants in the intervention group)**

- Question. What were your experiences with the remote coaching intervention?

Affective attitude

- Question. How did you feel about the remote coaching intervention?
- Question. How did you experience the personal contact with the coach?

Self-efficacy

- Question. To what extent were you able to follow the advices given during the remote coaching?
- Question. To what extent did you feel confident about your capability to follow the advices given during the remote coaching?

Perceived effectiveness

- Question. To what extent did you perceive the remote coaching intervention to be effective/beneficial?
- Question. To what extent did the remote coaching intervention help you with PA maintenance? In which way?

Ethicality

- Question. What were your expectations about the remote coaching intervention?
- Question. To what extent did the remote coaching intervention match with your expectations?

Intervention coherence

- Question. Could you explain what the aim of the remote coaching intervention was?

Burden

- Question. To what extent did you experience the remote coaching intervention as a burden?

Opportunity costs

- Question. To what extent did you have to cancel/reschedule other activities to participate in the remote coaching intervention?
